# Spatial and temporal changes in bird assemblages in forest fragments in an eastern Amazonian savannah

**DOI:** 10.1002/ece3.700

**Published:** 2013-08-06

**Authors:** Renato Cintra, William E Magnusson, Ana Albernaz

**Affiliations:** 1 Coordenação de Biodiversidade (CBio), Instituto Nacional de Pesquisas da Amazônia (INPA)69067-375, Manaus, Amazonas, Brasil; 2 Museu Paraense Emílio GoeldiBelém, Para, Brasil

**Keywords:** Brazilian Amazonia, forest bird assemblage, old-growth forest fragments, savannah

## Abstract

We investigated the effects of forest fragmentation on bird assemblages in an Amazonian savannah landscape with forest fragments that have been isolated for more than 100 years. The study was conducted in areas surrounding the village of Alter do Chão (2°31'S, 55°00'W), Santarém, Brazil. Bird surveys and measurements of tree density were undertaken in 25 areas, with 19 plots in forest fragments of different sizes and six in an area of continuous forest. Data on forest-fragment size, perimeter, and isolation were obtained from a georeferenced satellite image. Variation in number of bird species recorded per plot was not related to vegetation structure (tree density). The number of bird species recorded per plot increased significantly only with fragment area, but was not influenced by fragment shape or degree of isolation, even when considering species from the savannah matrix in the analysis. Fragments had fewer rare species. Multivariate ordination analyses (multiple dimensional scaling, [MDS]) indicated that bird species composition changed along a gradient from small to large forest fragments and continuous-forest areas. In the Amazonian savannah landscapes of Alter do Chão, the organization and composition of bird assemblages in forest fragments are affected by local long-term forest-fragmentation processes. Differences in the number of bird species recorded per plot and assemblage composition between forest fragments and continuous forest were not influenced by forest structure, suggesting that the observed patterns in species composition result from the effects of fragmentation per se rather than from preexisting differences in vegetation structure between sites. Nevertheless, despite their long history of isolation, the forest fragments still preserve a large proportion (on average 80%) of the avifauna found in continuous-forest areas. The fragments at Alter do Chão are surrounded by natural (rather than planted) grassland, with many trees in the savannah matrix and the landscape has vast areas covered by forest, which may have helped to ameliorate the influences of forest fragmentation.

## Introduction

Deforestation of tropical rain forests is currently one of the greatest threats to global biodiversity. Deforestation results in habitat loss, degradation, and fragmentation of a continuous landscape formerly covered by undisturbed forests. It has been advocated as one of the most negative effects produced by humans, leading many organisms to local extinction and reducing biological diversity. Many studies have demonstrated local reduction in diversity of mammals, birds, frogs, and ants, although there have also been recorded increases in local diversity of butterflies and hummingbirds (Laurance et al. 2002).

Bird species may respond to fragmentation in ways that are very different from other vertebrates. Previous studies have identified differences in vulnerability to extinction among species or groups of species. For example, some groups or guilds, such as insectivores, seem to be insensitive; although they were the first to disappear after fragmentation, they were also the first to return. Most frugivore species disappear from forest fragments after their isolation and few persist in small fragments surrounded by secondary growth forest. Nectarivorous birds are much less vulnerable, showing no clear declines in density due to forest fragmentation (Bierregaard and Stouffer 1997).

Most studies on the effects of forest fragmentation have been undertaken in habitat patches that were recently isolated by human activities, such as islands in dams (Terborgh et al. 1997) or forest patches isolated by cattle pasture and secondary forests (Laurance et al. 2002), and bird species may disappear after long periods of fragmentation even in large forest patches (Sigel et al. 2006). However, fragments can also be created by historical processes, such as the transition from forest to savannah. These processes, which can take hundreds to thousands of years, can isolate forest remnants at timescales much longer than those with drivers only related to human activities.

In northern Brazil, Amazonian-type savannahs occur in many areas within the Amazon rainforest biome (Pires and Prance 1985). In the municipality of Santarem, near the village of Alter do Chão, large areas of savannah isolate many forest fragments from the surrounding continuous forest. The area has a marked dry season, the savannahs often burn, and the landscape of forest fragments within the savannah matrix has probably existed for at least 4000 years. Natural processes, such as the contraction of savannas in South America, have occurred in the last 10,000 years (see Haffer 1969; Pennington et al. 2000). In the middle of the nineteenth century, Henry Bates commented on the occurrence of forest fragments isolated by savannah near Santarém and Alter do Chão (Bates 1892). The entire region was probably covered by forest about 4000 years ago (Sanaiotti et al. 2002; Toledo and Bush 2007; Costa et al. 2009; Roosevelt 2009). The forest fragments and savannah may represent the remainder of a postulated Pleistocene savannah, or were produced by Amerindian activities in the area (Vanzolini and Williams 1970; Costa et al. 2009), the origins of which are not clear. Most of this region, located between the Tapajós and Amazon Rivers, is covered by evergreen and semideciduous forests and large patches of savannah similar to those of the Cerrado vegetation in the plateaus of Central Brazil (Pires and Prance 1985). The soil characteristics and topographical conditions are similar among the forest fragments, which vary in size, shape, and isolation (distance to continuous forest). Although the forest patches have been isolated for long periods, the patches are separated by distances less than 1 km, and the dynamics of colonization and local extinction continue to the present.

Bird assemblages of the forest fragments could be subsets of the species pool present in the surrounding continuous forest. However, they could also result from a long, persistent, and dynamic process of fragment colonization from the nearer and larger forest fragments or from the neighboring continuous forest. If forest-fragment species composition was a function of colonization from adjacent areas of continuous forest, then size and isolation of forest fragments would be expected to influence species richness (total number of species that use a plot) and composition, as predicted by island biogeography theory (MacArthur and Wilson 1967; Cintra et al. 2007). Therefore, forest fragments near to one another and of a similar size should have similar species composition.

Bird assemblages in forest fragments may be affected by the period of time that has elapsed since isolation, the size and shape of the fragments, the quality of the fragments (e.g., variation in forest structure), and the degree of isolation or distance to the nearest continuous forest. Variation in forest structure may affect the bird assemblages in fragments and the continuous forest, independently of the effects of fragmentation, and differences in bird assemblages could be due to preexisting differences in vegetation between the fragmented- and continuous-forest landscapes. Also, edge effects influence tropical secondary forest bird assemblages (Banks-Leite et al. 2010). As all our plots were placed starting from the edge to the center of the forest fragments, the proportion of the edge was similar in all fragments studied. We used the shapes of the fragments (which represent the relative susceptibility to edge effects) in analyses, together with other forest-fragment attributes, to evaluate their effects on the bird assemblages.

The abundance of aggressive bird species could potentially affect the number of bird species recorded per plot and species composition in isolated forest fragments. Two opportunistic, territorial and relatively aggressive bird species, the Antshrike *Thamnophilus stictocephalus*, which is wide spread in the area (Dantas et al. 2005), and the Buff-breasted Wren, *Cantorchilus leucotis*, were recorded in 89% and 95% of the fragments, respectively, and in 100% and 83% of continuous-forests sites. These birds had relatively high abundance in most sites and could potentially affect the occurrences of other bird species and consequently affect the number of bird species recorded per plot or composition because of the negative influence they exert in isolated forest fragments. We have no information about this type of relationship in studies on the effects on birds of forest fragmentation in the Amazon or elsewhere in Brazil, but effects of dominant species have been found for ant assemblages in our study area (Vasconcelos et al. 2006).

There are many studies on birds of Amazonian savannahs which produced lists of species that are useful to describe distributional patterns (see Lees et al. 2013). However, there are still few ecological studies on how forest environments can influence changes in the number of bird species recorded per plot and composition of bird assemblages in forests and savannahs in Amazonian savannah landscapes (but see Cintra et al. 2007; Cintra and Naka 2012). Here, we present the first study of bird assemblages from ancient forest fragments in the savannahs of Alter do Chão near Santarem in eastern Amazonian Brazil.

We tested the effect of forest fragmentation on bird species richness (indexed by the number of bird species recorded per plot), abundance, and assemblage composition; using data based on transect surveys. Specifically, we tested the hypothesis that the forest-fragment characteristics (area, shape) and location (isolation), the spatial variation in the forest structure (tree density), and some landscape features (savanna habitat matrix) influence the occurrence, abundance, and distribution of bird species. Because among-year differences can also determine the spatial distribution of bird species richness in tropical forests, we also tested the effect of seasonality on the avifauna.

Some species more frequently forage and nest in areas with higher densities of trees and shrubs, whereas others use more open areas with less abundant trees. Because fragment features, such as size, shape, and isolation from other fragments also vary spatially, bird species composition may also differ across many fragments over a large area, probably following gradients of spatial variation in ecological and environmental factors (Cintra and Naka 2012).

Our expectation was that forest-fragment shape and isolation in the savannas of Alter do Chão would not influence bird assemblages, as most tend to be rounded to elliptical and not very isolated from each other. However, because variation in fragment area may be independent of fragment shape, and larger fragments may support higher bird species richness, we expected influences on bird assemblages and the relative occurrence of trophic guilds.

Using standardized protocols (transect surveys), we examined the following general question: does the avifauna of the forest fragments differ in the number of bird species recorded per plot and composition from that of the adjacent areas of continuous forest? Using data collected in 1999 and 2000, we compared the number of bird species recorded per plot and species composition between isolated forest fragments and sites in the continuous forest, and evaluated how forest-fragment size and shape, the degree of isolation from the continuous forest, and forest structure (tree density) affected the bird assemblages and their trophic guilds.

## Methods

The study was conducted in a 29,000 ha area near the village of Alter do Chão, in Santarém (2°31'S, 55°00'W), Pará State, Brazil. The mean annual temperature between 1999 and 2000 in Santarém was 27.5°C, two-thirds of the annual rainfall (mean = 2192 mm between 1999 and 2000) falls between January and June, with a pronounced dry season from June to November. The soils in the area are mainly sandy. The area is covered by two main vegetation types: Amazonian savannah and semideciduous forest in patches within the savannah matrix and in continuous forest surrounding the savannahs (Pires and Prance 1985). The vegetation in the savannah areas is dominated by a herbaceous stratum composed principally of the grasses *Paspalum carinatum* and *Trachypogon plumosus*, interspersed with small patches of trees and shrubs. The semidecidous forests have a relatively open understorey and contain tree species common in the region, such as *Dalbergia spruceana* (Fabaceae), *Myrcia fallax* (Myrtaceae), *Mezilaurus itauba* (Lauraceae), *Tabebuia serratifolia* (Bignoniaceae), *Lecythis pisonis* (Lecythidaceae), *Himatanthus articulatus* (Apocynaceae), and *Eschweilera obversa* (Lecythidaceae) (Sanaiotti et al. 2002).

### Estimates of fragment sizes and distances from continuous forest

**Figure 1:**
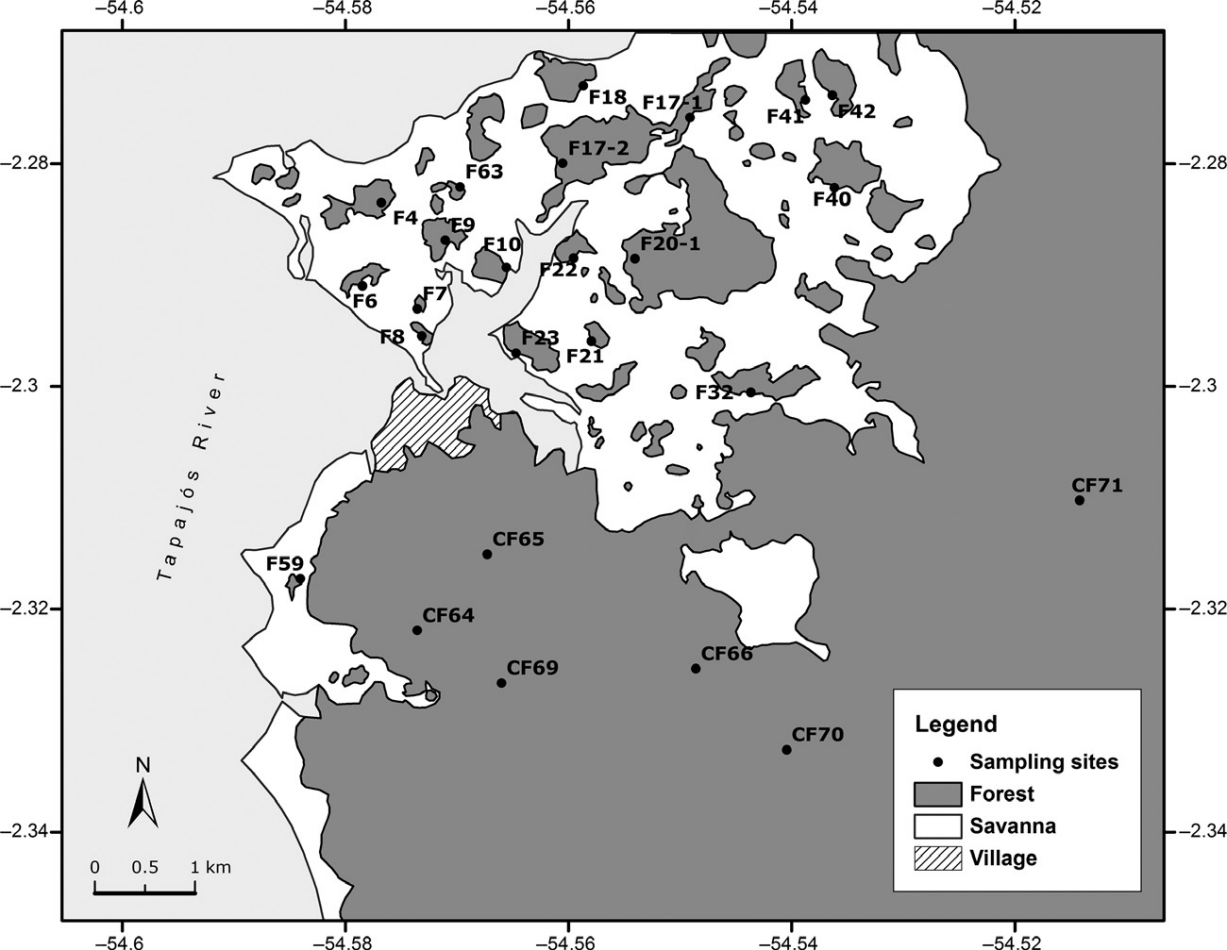
Study area showing the forest fragments in the savannah landscape, and the surrounding continuous forest, Pará, eastern Amazonia, Brazil.

The sizes of the fragments were calculated using the areas of polygons created after the digitalization of the forest areas taken from a satellite image (Landsat TM5, Image orbital location: 227-62 ; Santarem, Alter-do-Chão, Pará, Brazil), which was georeferenced from 12 control points using global position system, USA (Margelin) and digitalized through the CAMRIS Program. The program was used to calculate the size of the forest fragments. For each of the six areas located in the continuous forest, which were located 1–10 km from one another (Fig. 1), we arbitrarily used 500 ha as the estimated area. Because forest fragments varied from 2.4 ha to 360.4 ha, we log transformed the values of the fragment area before running the analysis. The distance from each forest fragment to the nearest forest fragment (hereafter distance to FF), to the continuous forest (hereafter distance to CF), fragment width (km), shape, and area (ha) were estimated on the satellite image using the “rule” tool available in the program Arc View 3.2 (ESRI 1996). Data on the location and characteristics of the forest fragments are given in Table 1.

**Table 1:** Characteristics of the forest fragments and continuous forest of Alter do Chão, Pará, eastern Amazonia, Brazil

Site	Latitude	Longitude	Length (km)	Width (km)	Shape	Area (ha)	Log area	Dist. Fo (km)	Dist. Fg. (km)	Trees
F4	−54.96292	−2.47232	0.76	0.61	1.35	31.5	3.44999	8.134	0.39	421
F6	−54.96450	−2.48375	0.72	0.19	1.93	8.5	2.14007	11.023	0.762	1020
F7	−54.95524	−2.48765	0.23	0.15	1.21	2.4	0.87547	10.536	0.507	234
F8	−54.95488	−2.49195	0.41	0.13	1.64	3.8	1.335	10.824	0.507	753
F9	−54.95219	−2.47826	0.72	0.66	1.94	26.3	3.26957	7.779	0.551	923
F10	−54.94630	−2.48304	0.58	0.57	1.4	22.4	3.10906	6.918	0.5739	780
F17-1	−54.94989	−2.47089	3.08	1.01	2.2	37.8	3.63231	3.396	0.544	216
F17-2	−54.93402	−2.46683	1.05	0.67	1.6	152	5.02388	4.861	0.819	871
F18	−54.91492	−2.45923	2.59	2.54	1.47	46.7	3.84374	5.325	0.43	363
F20-1	−54.93324	−2.45545	2.59	2.54	1.9	360.4	5.88721	3.538	0.54	214
F21	−54.89251	−2.46923	0.46	0.31	1.22	10.6	2.36085	2.408	0.593	169
F22	−54.91916	−2.48508	0.67	0.35	1.54	14.7	2.68785	4.62	0.72	561
F23	−54.92392	−2.48115	1.03	0.42	1.6	39.6	3.67883	3.364	1.07	341
F32	−54.93384	−2.47996	1.77	0.53	1.73	59.7	4.08933	0.421	0.959	151
F40	−54.91359	−2.49530	1.14	0.83	1.66	66.4	4.1957	2.235	0.635	111
F41	−54.96405	−2.54306	0.99	0.45	1.75	28.2	3.33932	1.569	0.495	172
F42	−54.97554	−2.53140	1.16	0.52	1.58	42.8	3.75654	0.79	0.63	147
F59	−54.89073	−2.45586	0.48	0.24	2.05	6	1.79176	0.305	0.305	344
F63	−54.90245	−2.49870	3.08	1.01	1.68	7.5	2.0149	6.577	0.3826	928
CF64	−54.95714	−2.54107	–	–	–	–	–	–	–	143
CF65	−54.95477	−2.52992	–	–	–	–	–	–	–	199
CF66	−54.92504	−2.53578	–	–	–	–	–	–	–	157
CF69	−54.94904	−2.54674	–	–	–	–	–	–	–	235
CF70	−54.89786	−2.54858	–	–	–	–	–	–	–	204
CF71	−54.51268	−2.30103	–	–	–	–	–	–	–	380

Codes: *F*, forest fragments; CF, continuous forest; Dist. Fo, Distance to the nearest continuous forest; Dist. Fg, Distance to the nearest forest fragment; Trees, number of trees.

### Vegetation structure

Trees were marked and measured along the four 2 × 250 m transects, covering an area of 0.2 ha in each of the 25 areas where birds were surveyed. Data on trees were recorded by INPA (Biodiversity Department) technicians. Trees and saplings with diameter at breast height (DBH) >1.6 cm within 1 m of either side of the transects were measured (total area sampled = 0.2 ha). The total number of trees and saplings with DBH greater than 1.6 cm/0.2 ha was used as an index of vegetation structure because this was highly correlated with other indices of vegetation structure, such as the principal components based on numbers in different size classes. This index of vegetation structure was used because it is simpler to interpret. The number of trees used in the analysis is shown in Table 1.

### Sampling protocol and bird surveys

During the bird surveys, visual counts were carried out in 25 plots of 250 × 150 m (3.75 ha), with 19 plots in forest fragments of different sizes. Therefore, our sample unit was a plot of 3.75 ha. The forest fragments were distributed within a savannah landscape surrounded by continuous evergreen and semideciduous forest. Distances between fragments varied from 0.8 to 12.5 km. Six forest plots (each of 3.75 ha) were located in areas of continuous forest, with a minimum of 1 km from one another (Fig. 1). Parallel transects were established in each of six randomly selected sites in continuous forest and in 19 sites in fragments (Fig. 1). In each site, there were four transects which were 250 m long and 50 m apart and were marked at 50-m intervals with colored plastic numbered flags nailed to tree trunks. Transects in forest fragments were perpendicular to the forest edge, running from the edge toward the interior of the fragment. Forest fragments ranged from 2.4 to 360.4 ha, but only two were larger than 100 ha. The two largest forest fragments were sampled at two locations, but in the analysis we pooled data for the two sets of transects (see below) in each fragment and used the resulting mean values. We undertook the surveys during the peak season for bird breeding activities in the area (see Sanaiotti and Cintra 2001), so patterns of abundance could be slightly different in other seasons. Surveys were undertaken in September 1999 and repeated in November 2000.

Bird surveys were conducted by Renato Cintra, who has been working in the area for more than 15 years and is familiar with the bird species. The bird surveys within each plot consisted of the following protocol (sampling effort): walking slowly (about 1.5 km/h) along the two parallel transects in the middle of the plot separated by 50 m from one another. The observer walked in one transect and then walked back in the opposite direction along the other transect, stopping for 2 min every 50 m (six observation points along each transect) and recording all birds seen in a strip 30 m long and 5 m wide, extending from the observation point toward the plot border. Birds were viewed with 10 × 30 Nikon binoculars and a 30–50 zoom Nikon telescope. Individuals heard while walking between observation points were not recorded. The short time spent at each observation point minimized the risk of counting the same bird twice. A Sony microcassette tape recorder was also used to register bird calls and only used to help identify inconspicuous species. Surveys were performed daily between 0600 h and 0900 h and two plots were surveyed per day. On the same day, between 1500 h and 1800 h, the same two plots were surveyed again following the same protocol. Categorization into feeding guilds (omnivores–insectivores, hereafter omnivores, and frugivores) was based on direct field observations of bird feeding activities, complemented by information from a study on the diet of Brazilian birds by Schubart et al. (1965). We did not analyze nectarivores separately because we detected a low number of species.

### Analyses

#### Differences in bird species richness between forest fragments and continuous forest

To test the hypothesis that the species richness in the fragments is lower than that in continuous forest, the numbers of species found in the forest fragments and continuous forest (all sites combined) were compared using sample-based rarefactions curves. These curves were built using the program EstimateS (Colwell 2005). Differences in the total number of species, in the number of species that also occurred in the savannah matrix, in the number of rare species, and differences between forest fragments and the continuous forest were compared using *t*-tests.

We use multiple linear models using the number of bird species recorded per plot as a dependent variable against the independent variables forest-fragment size, distance to FF, distance to CF, and forest-fragment shape. However, when fragment shape was included, the data from sites in the continuous forest could not be included in the analysis.

#### Effects of vegetation structure (tree density) on bird richness

To determine whether the vegetation structure affected the number of bird species per plot, an analysis of covariance was used to compare the number of bird species recorded per plot between forest fragments and continuous-forest areas, taking into account tree density as a covariate.

#### Effects of aggressive bird species on bird-assemblage composition

To test the hypothesis that the aggressive bird species *C. leucotis* and *T. stictocephalus* negatively influence the number of bird species recorded per plot and composition in isolated forest fragments, we analyzed the correlation between *T. stictocephalus* and *C. leucotis* abundances (total number of birds recorded) and the number of bird species recorded per plot, and bird species composition (using a single nonmetric multiple dimensional scaling, [NMDS] ordination axis with a one-dimensional solution, see below). Statistical tests were undertaken in the Systat program (Wilkinson 2007).

#### Differences in bird-assemblage composition among forest fragments of different sizes, and between fragments and continuous forest

To determine whether bird-assemblage composition differed among forest fragments of different sizes, and between fragments and continuous forest, we used NMDS, hereafter called MDS, to ordinate sites by their similarity in bird species composition using Bray–Curtis and Sorensen indices for quantitative (species abundance by sites) and qualitative (species presence–absence by site) matrices. The indices and MDS are available in the program PC-ORD (McCune and Mefford 1999). To evaluate differences in bird species composition (expressed as MDS ordination scores) between forest fragments and continuous forest, we constructed quantitative and qualitative MDS ordinations for the whole community, for omnivores, and for frugivores (see also Appendix S2). We included the 19 sites in the forest fragments and the six sites in the continuous forest in all ordinations. An additional ordination was run after removing bird species from the data matrix that also occurred in the savannah areas.

An a posteriori Pillai's Trace test was used to investigate whether multivariate analysis of variance (MANOVA) (Appendix S2) would reveal significant differences among sites, and to evaluate the effects of forest-fragment characteristics (size, shape, and isolation from the nearest fragment and from areas of continuous forest) and forest structure (tree density) on bird-assemblage composition (using multiple linear models and Pillai's trace tests). Pillai's trace statistics have been shown to be less sensitive to deviations from assumptions than other multivariate inferential statistics (Borg and Groenen 1997).

We also ran three MDSs for the bird assemblages only from the 19 forest fragments, using only qualitative matrices (bird species presence/absence data), with one MDS including the entire sampled bird pool, one the guild of omnivores, and another including only frugivores. In these three ordinations, each with a one-dimensional solution, only data from forest fragments were used. The use of a single ordination axis allowed us to examine the partial effects of fragment area, distance to FF, distance to CF, and fragment shape on bird species composition by means of multiple linear models. The resulting MDS ordination scores (both from quantitative and qualitative matrices) were also used to compare bird-assemblage composition among the forest fragments between 1999 and 2000.

Multiple linear models available in the Systat program (Wilkinson 2007) were used to investigate how forest-fragment size, distance to FF, distance to CF, and forest-fragment shape affect the number of bird species recorded per plot and to examine how these same four variables influence variation in bird-assemblage composition. Data on forest-fragment area and distance to FF were log 10 transformed prior to analysis to meet the assumptions of normality. We used partial residual plots, available in the R program (http://www.r-project.org), to evaluate the independent effects of predictor variables. We also used R to investigate possible linear relationships among predictor variables, estimating the variance inflation factor resulting from multicolinearity.

#### Correlations between the geographic locations and attributes of the forest fragments

To evaluate the existence of correlations between the geographic locations and attributes of the forest fragments, we used Mantel tests, implemented in the PC-ORD program, to investigate spatial correlation among variables, or the significance of relationships between assemblage matrices for similarity and distance between forest fragments. For this, we used the most complete data set of bird assemblages from the 19 forest fragments, or only the qualitative matrix of bird species by sites from the year 2000 without including species from the savannah areas. We also used Mantel tests to evaluate correlations between the geographic location of the forest fragments for variables such as forest-fragment area, shape, distance to the nearest forest fragment, distance to continuous forest, and tree density.

#### Differences in bird assemblages between 1999 and 2000

To investigate whether the rate of changes in the bird community in a given forest fragment was consistent from 1999 to 2000, we first placed the matrices from 1999 to 2000 together with the corresponding bird species and forest fragments and used a Bray–Curtis dissimilarity (for quantitative data) or a Sørensen distance measure (for qualitative data) to describe dissimilarity between sites in bird abundance and occurrence between years. The resulting MDS ordination scores values were then used as dependent variables in multiple linear models, using as independent variables fragment size, distance to FF, distance to CF, and forest-fragment shape.

## Results

### Differences in bird species richness between forest fragments and continuous forest

We only had three areas prepared in 1999 for sampling in the continuous forest, and therefore most of the results and the list of species (Appendix S2) are presented only for the year 2000; however, we also compared the forest-fragment bird assemblages between 1999 and 2000.

In 2000, we made 1731 registers (1165 in the forest fragments and 476 in areas of continuous forest) of 144 bird species for all 25 forest sites surveyed, including 19 forest fragments and six plots in the continuous-forest area. We recorded 124 bird species in the forest fragments and 113 in the continuous-forest sites. Twenty-two bird species (15.1%) that were recorded in the continuous-forest sites were not recorded in the forest fragments, and 33 bird species (22.6%) were recorded only in the forest fragments. Twenty-four (16.4%) bird species were recorded once in the forest fragments and 31 (21.2%) species were recorded only once in the continuous-forest sites. Thirty-two species that occurred in the forest (including sites of both forest fragments and continuous forest) also occurred in the savannah matrix.

Among the most abundant species across all assemblages were the following four species: the Buff-breasted Wren, *C. leucotis* (145 of records; 8.8% of all records), the Antshrike *T. stictocephalus* (107; 6.5%), the Blue-backed Manakin, *Chiroxiphia pareola* (73; 4.4%), and the White-fringed Antwren, *Formicivora grisea* (71; 4.3%).

Site-to-site variation in the number of bird species recorded per plot was relatively large. For the continuous-forest areas, the number of bird species recorded per plot ranged from 38 (sites 71 and 65) to 57 (site 69), and abundance from 57 (site 70) to 107 records (site 69). In the forest fragments, the number of bird species recorded per plot ranged from 23 (site 22) to 47 (site 17-2) and bird abundance from 32 (site 20) to 89 records (sites 41 and 42). The most frequently encountered bird families considering all 25 areas were Tyrannidae, with 25 species (17.4%), and Thraupidae, with 12 species (8.3%).

**Figure 2:**
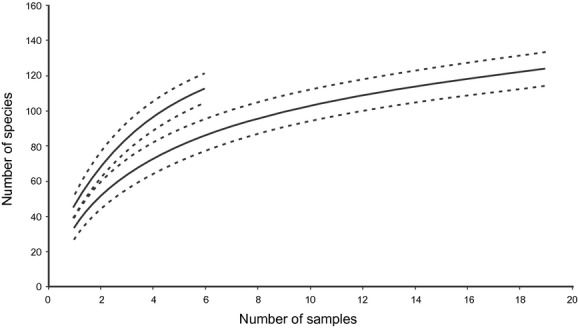
Sample-based rarefaction curves of the number of bird species in continuous-forest areas (upper continuous curve) and forest fragments (lower curve). Dotted curves show the lower and upper 95% confidence intervals for each continuous curve.

Differences in the number of bird species recorded per plot between forest fragments and continuous forest were detected when combining data from all sites. The total number of species found in the continuous forest was similar to the number found in the forest fragments, but sample-based rarefaction curves (Fig. 2) indicated that the total number of species found in the continuous forest (113 species) was significantly greater than the number expected to be found in the forest fragments with the same sampling effort (86.5 ± 4.57 species in six samples).

**Figure 3:**
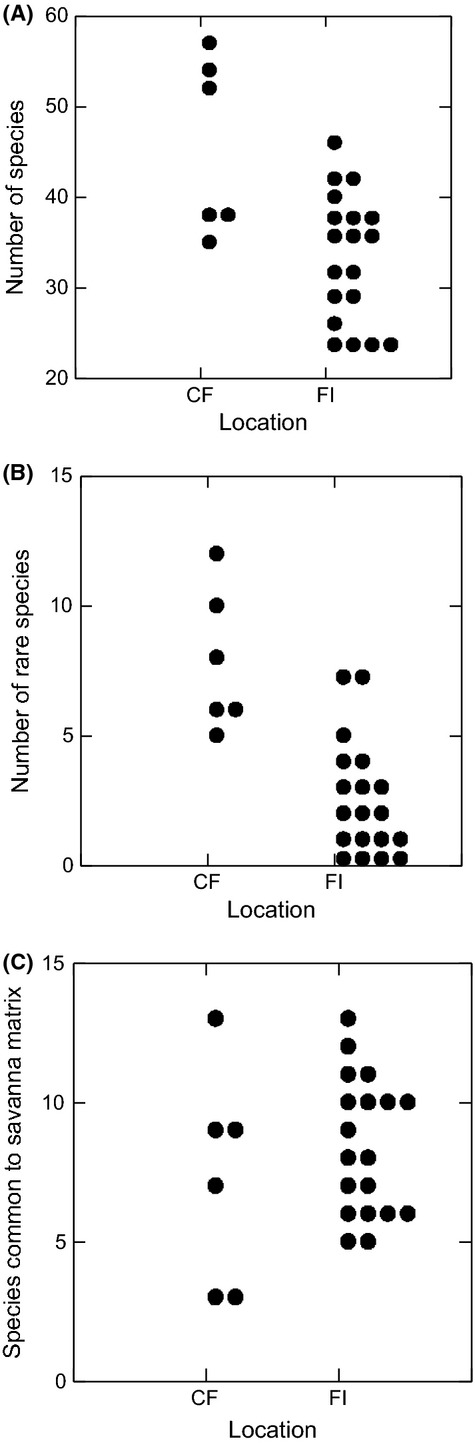
Differences between continuous forest (CF) and forest fragment (FI): (A) total number of species; (B) species recorded in fewer than four forest sites (rare species); (C) number of species common to the savannah matrix.

The forest fragments had significantly fewer rare species than were found in the continuous forest (*t* = −4.447, d*f* = 23, *P* < 0001). However, forest fragments and continuous forest had similar numbers of species (*t* = 0.821, d*f* = 23, *P* = 0.420) that are known also to occur in the savannah matrix (Fig. 3).

We also evaluated variation in the number of bird species recorded per plot in relation to forest-fragment area, considering only the forest fragments and pooling data from 1999 to 2000, in two separate analyses, one with and the other without bird species from the savannah matrix. The number of bird species recorded per plot increased significantly with fragment size in the analysis including the species from the savannah matrix (*r*^*2*^ = 0.490; *F* = 19.232; *P* = 0.0001) and that without species from the savannah (*r*^2^ = 0.674; *F* = 41.345; *P* = 0.0001).

### Effects of vegetation structure (tree density) on bird richness

Variation in the number of bird species recorded per plot was not related to variation in tree density when considering the whole species pool (Ancova, *F*_1,22_ = 0.672, *P* = 0.421; for omnivores, *F*_1,22_ = 1.320, *P* = 0.263; and frugivores, *F*_1,22_ = 0.001, *P* = 0.972), nor was there a significant interaction between the effects of habitat type (forest fragments vs. continuous forest) and tree density on the number of bird species recorded per plot.

### Effects of aggressive bird species on bird-assemblage composition

Variation in numbers of the aggressive and common antbird *T. stictocephalus* was not significantly related to the number of bird species recorded per plot (correlation analysis, *r* = 0.286, *P* = 0.166, *n* = 25) or bird species composition (*r* = −0.087, *P* = 0.680, *n* = 25). The numbers of records of the other common aggressive species, the wren *C. leucotis*, were also not related to the number of bird species recorded per plot (*r* = 0.095, *P* = 0.650, *n* = 25) or bird species composition (*r* = 0.181, *P* = 0.387, *n* = 25).

### Differences in bird-assemblage composition among forest fragments of different sizes and between fragments and continuous forest

**Figure 4:**
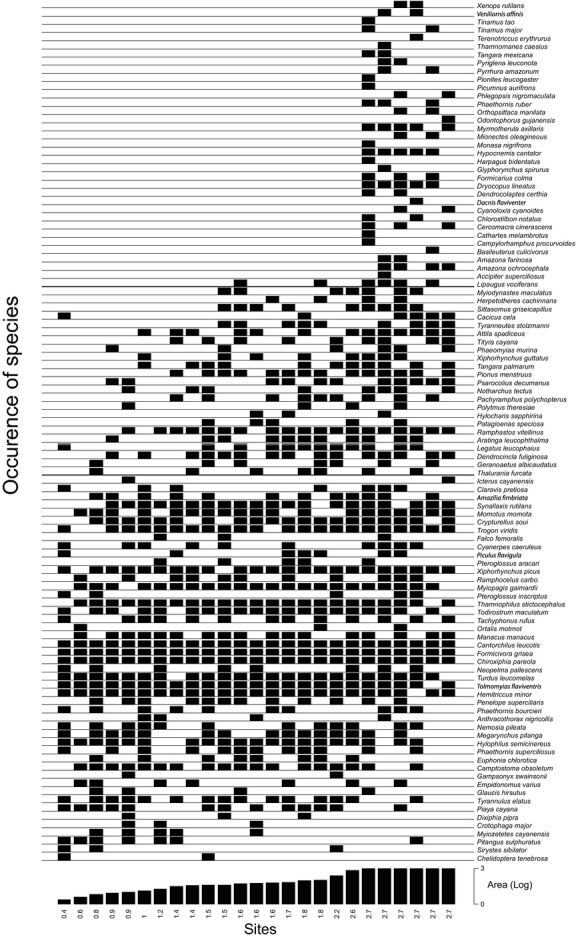
Scatter plot of the relationship between fragment area and number of bird species recorded per plot. The figure is only for qualitative (presence/absence) values.

**Figure 5:**
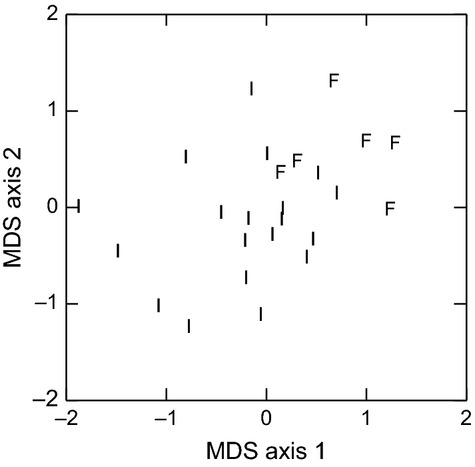
Multidimensional scaling (MDS) ordination in two dimensions of the bird assemblages from forest fragments (I) or continuous-forest sites (*F*). Ordination was based on species presence–absence data.

In general, bird species composition differed in sites in forest fragments from those in continuous forest (Fig. 4 and 5). We found significant differences in the composition of bird species (qualitative and quantitative data) along the forest-fragment area gradient. Extremes of the forest-fragment area gradient (small and large) were separated well along axis one, whereas samples from medium-sized fragments were intermediate (Fig. 4). The bird assemblages in the fragments were subsets of those in the continuous forest. Several species were recorded exclusively in the continuous forest (upper part of Fig. 4), others occurred in the continuous forest and also in the forest fragments (middle and lower part of Fig. 4). No species were common in fragments but absent from the continuous-forest plots. The same pattern held when the species composition was compared among the forest-fragment sizes (small, medium, and large) and between fragments and areas in the continuous forest (see Appendix S2).

### Autocorrelation of the forest-fragment attributes

We found statistically insignificant correlations between the geographic location of the forest fragments for variables such as forest-fragment area (Mantel test, *r* = −0.063, *t* = −0.365, *P* = 0.715), forest-fragment shape (*r* = −0.062, *t* = −0.478, *P* = 0.633), distance to the nearest-neighbor forest fragment (*r* = −0.1187, *t* = −0.822, *P* = 0.411), distance to continuous forest (*r* = 0.153, *t* = 1.387, *P* = 0.166), and tree density (*r* = −0.080, *t* = −0.799, *P* = 0.425). Because these variables had very low Mantel test “*r*-values”, and were so weakly correlated with location (geographical coordinates), we included them as independent variables in the multiple linear models.

The relationship between distances in the qualitative bird-assemblage composition (without savannah species) and distances between forest fragments was not significant (Mantel test, *r* = 0.104; *t* = 0.727, *P* = 0.467), indicating that bird species composition of the forest fragments was not affected by spatial proximity.

### Differences in bird assemblages between 1999 and 2000

**Figure 6:**
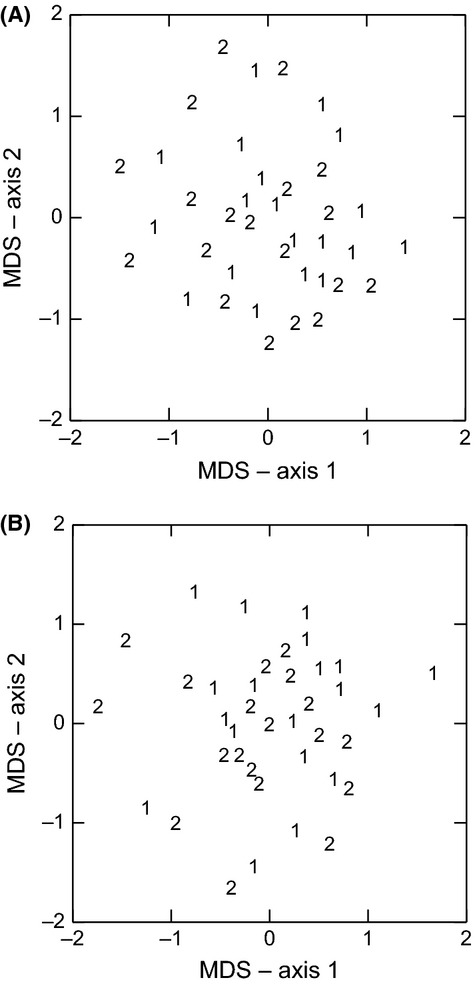
Multidimensional scaling (MDS) ordination in two dimensions of the bird species composition from forest fragments in 1999 (1) and from the same forest fragments in 2000 (2). Ordination was based on species/presence absence data and using the same bird species that occurred in both years, including the species that also occurred in the savannah matrix. The upper graph includes 100 species (including those that also occurred in the savannah matrix; the lower graph show the results of MDS ordination after removing the species that also occurred in the savannah matrix).

The bird species composition (using MDS ordination and a qualitative data matrix with the same 100 bird species that occurred only in the fragments in 1999 and in 2000) did not differ significantly between 1999 and 2000 (MANOVA, Pillai's trace test = 0.034, *F*_2,35_ = 0.616, *P* = 0.546), indicating that the species composition did not show strong year-to-year changes. Without the species that also occurred in the savannah matrix, the bird species composition also was not significantly different between 1999 and 2000 (MANOVA, Pillai's trace test = 0.099, *F*_2,35_ = 1.922; *P* = 0.161), indicating that, independent of the presence of savannah species in the assemblages, composition showed little change between years (Fig. 6). For both quantitative and qualitative analyses, bird species composition did not vary significantly in relation to fragment area, distance to nearest fragment, distance to continuous forest, or fragment shape (Appendix S2).

### Effects of forest fragmentation on number of bird species and on variation in bird-assemblage composition

Results from multiple linear models indicated that the number of bird species from the total species pool (and for omnivores and frugivores) in the fragments was significantly affected by forest-fragment area (Table 2, only results for the whole species pool are shown excluding bird species of savanna from the analysis. The bird species composition was also significantly influenced by forest-fragment size; see below).

**Table 2:** Results of multiple linear regression analysis for the effects of forest-fragment area (log transformed), distance to the nearest forest fragment (FF), distance to continuous forest (CF) (log transformed), and fragment shape on the number of bird species recorded per plot and species composition (*n* = 19 forest fragments), not including bird species from the savannah

Dependent variable	Explanatory variable	Regression coefficient	Standardized coefficient	*t*	*P*
Number of species	Area	2.613	0.558	2.443	0.028
	Distance to FF	−3.933	−0.132	−0.649	0.527
	Distance to CF	−0.504	−0.301	−1.381	0.189
	Shape	−1.462	−0.067	−0.340	0.739
Species composition	Area	1.201	0.636	2.639	0.019
	Distance to FF	−0.557	−0.106	−0.455	0.656
	Distance to CF	0.249	0.111	0.482	0.637
	Shape	−0.337	−0.088	−0.380	0.710

The general pattern of the effects of forest-fragment characteristics on bird species composition (using the MDS one-dimensional solution, resulting from the matrix of species presence/absence) was consistent for the three bird groups considered. The results of multiple linear models for the whole species pool (see also Appendix S2), the omnivore–insectivores and the frugivores were significantly affected by fragment size (*P* = 0.015, *P* = 0.023, *P* = 0.034, respectively), but not by distance to the continuous forest (*P* = 0.637, *P* = 0.861, *P* = 0.107, respectively), distance to the nearest forest fragment (*P* = 0.656, *P* = 0.433, *P* = 0.282, respectively), or fragment shape (*P* = 0.710, *P* = 0.687, *P* = 0.730, respectively). The full multiple linear models for the whole species pool, omnivores, and frugivores explained respectively 58, 56, and 60% of the variance in the ordination scores.

**Figure 7:**
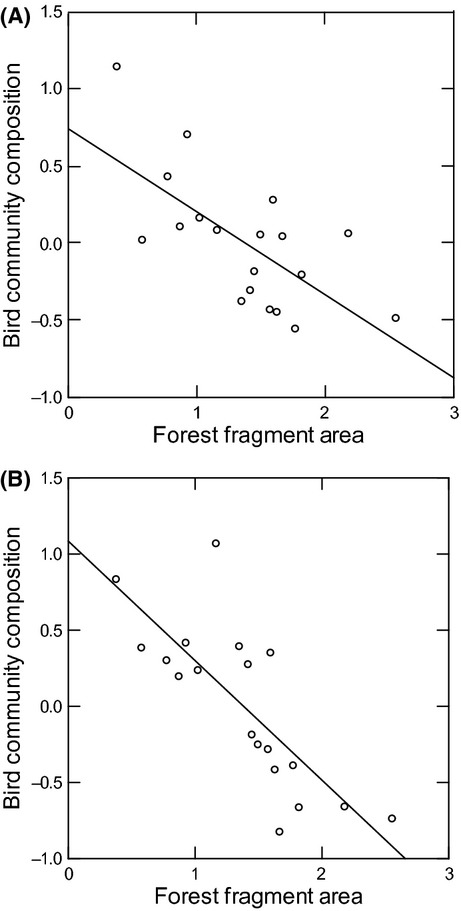
Relationship between bird species composition (run in one dimension in the MDS ordination analysis; based on qualitative data and the presence/absence matrix) and forest-fragment area (log 10 transformed). Upper graph (data from 1999) and lower graph (data from 2000).

Without species that were also found in the savannah (32 species; see Appendix S1), the forest-fragment area also significantly affected the number of bird species recorded per plot and species composition (Table 2). Species composition changed monotonically as the forest-fragment area increased (Fig. 7).

## Discussion

The results of this study clearly indicate that, in the Amazonian savannah/forest landscapes of Alter do Chão, the organization and composition of bird assemblages are affected by the local long-term processes of forest fragmentation. As found in other studies evaluating the effects of Amazonian forest fragmentation on bird assemblages, forest fragments had fewer rare bird species and the species present in the forest fragments tended to be a subset of those found in sites in the continuous forest. This study adds to the growing body of evidence suggesting that long-term forest fragmentation of tropical forest can affect the local composition of animal communities. In the same area, similar results were found for assemblages of ants (Vasconcelos et al. 2006), beetles (Louzada et al. 2010), forest lizards (Jr Carvalho et al. 2008), and bats (Bernard and Fenton 2002).

In general, differences in the number of bird species recorded per plot (including rare species) and species composition between forest fragments and continuous forest were not influenced by forest structure (tree density), suggesting that the observed pattern in species composition may result from the effects of increasing forest insularity (fragmentation) per se rather than due to preexisting differences in vegetation structure between those sites, or structural changes due to edge effects. In general, the number of species recorded per plot in the forest fragments was lower than that in the continuous-forest areas, and the bird assemblages in the fragments were subsets of those in the continuous forest.

Our results suggest that bird assemblages are affected by characteristics of the forest fragments independently of the bird guild considered. Fragment size was significantly related to the number of bird species recorded per plot and abundance even when sites from continuous forest were included. The significant variation in the number of bird species recorded per plot and bird abundance can be attributed to species turnover within the study area; not due to biogeographical or regional variation in the avifauna, but rather to local changes in the avian assemblages. Our surveys indicate that most bird species occur throughout the area (see Appendix S1). Therefore, we believe that the results indicate that birds are tracking differences in the landscape and in forest-fragment size. Even when considering only forest-fragment sites in the analysis, fragment area influenced the number of bird species recorded per plot and composition (Table 2). Furthermore, our results also suggest that the small, medium, and large forest fragments in the savannah of Alter do Chão contain very dynamic and variable assemblages. Quantitative and qualitative analyses indicated that the bird assemblages in the continuous forest were significantly different from those of the forest fragments, and this pattern held for the entire species pool, omnivore–insectivores, and frugivores.

This scenario may have resulted from long-term isolation of the forest fragments in the area, even though a few of them are located less than 1 km from the continuous forest. We still do not understand the mechanisms responsible for species loss in smaller forest fragments. Nest predation could be a major cause of species loss in forest fragments (Melo and Marini 1997). Although in our study area tree density seemed to be less important, vegetation may be a determining factor. In other areas of the Amazon region, tree assemblage structure and phenological dynamics vary spatially and temporally and can affect bird assemblages (Cintra and Naka 2012). In Africa, it has been demonstrated that changes in vegetation structure cause changes in bird-assemblage composition (Skowno and Bond 2003). In Madagascar, forest species are positively correlated with tree and shrub cover and, in small- and medium-size forest fragments, the loss of habitat structure and complexity due to tree and shrub removal, along with the food and nesting resources associated with them, could be the primary cause of bird species loss (Scott et al. 2006).

Distance to continuous forest did not influence the number of bird species recorded per plot or composition in forest fragments. However, more species tended to occur in fragments that were near forest than in those that were far from other forest areas, regardless of the size of fragments. This indicates that, in Alter do Chão, the surrounding continuous forest is a source of colonists for the fragments, as predicted by the theory of island biogeography (MacArthur and Wilson 1967; Cintra et al. 2007). The continuous forest, therefore, could maintain species in smaller and isolated forest fragments by a “rescue effect.” However, in our study area, the landscape has vast areas covered by forest, and many trees in the savannah matrix, which may buffer the local impacts of fragmentation.

The forest of Alter do Chão is not very disturbed and seems to be the same as it was centuries ago in terms of bird assemblages and vegetation structure (Sanaiotti et al. 2002). The forest fragments may have been isolated for 4000 years, and their bird assemblages are probably under a new and dynamic biological equilibrium. Some omnivores (species of the genus *Lepidocolaptes*, *Formicivora*, *Myiarchus*, *Elaenia*, *Nystalus*, *Turdus*, and others) marked with aluminum bands have been recaptured in the savannah areas for periods of more than 10 years (T. Sanaiotti and R. Cintra, unpubl. data). In the central Amazon, stronger effects of area have been shown compared to the effects of isolation, and some species can persist in small fragments for more than a decade (Laurance et al. 2002). However, because the birds usually move through shrubs, which are components of open savannahs, they could cross from one forest fragment to another and colonize other areas.

We have concentrated our analysis on data from a single period of the year because the area is drier and more seasonal than other areas of the Amazon region, and the temporal patterns appear similar when rare and abundant and/or only abundant species are considered. We believe that the species recorded in this study approximately represent the distribution of birds within the forest fragments and areas of continuous forest throughout the year. Most of the species (95%) present in the fragments in 1999 were also present in the same fragments in 2000, although the assemblages were variable.

Although the fragments were formed a long time ago (perhaps more than 4000 years), our results are based on short-term bird surveys (2 years) and address only broad-scale guild and assemblage responses based mainly on the number of bird species recorded per plot and composition. Therefore, our results should be interpreted with caution. In addition, the fragments and surrounding continuous forests are embedded in a much larger landscape, where large areas of continuous forest are still available and which have potentially minimized the local impacts of forest fragmentation on bird assemblages. Nevertheless, they indicate that complex assemblages can persist in forest fragments isolated for long periods.

## Supporting Information

**Appendix S1.** Bird species recorded in the areas of island and continuous forests of Alter-do-Chão, Para, and eastern Brazilian Amazonia (see methods; and codes at the end of this table).

**Appendix S2.** Analysis on bird guilds.
