# Supplementary material for: Spatial and temporal changes in bird assemblages in forest fragments in an eastern Amazonian savannah
Source: Ecol Evol. 2013 Aug 6;3(10):3249–62. doi: 10.1002/ece3.700 (PMC3797474; doi:10.1002/ece3.700)
Supplement: Supplementary file 1 [file ece30003-3249-SD1.doc]

Appendix S1. Bird species recorded in the areas of island and continuous forests of Alter-do-Chão, Para, and eastern Brazilian Amazonia (see methods; and codes at the end of this table).

* Scientific and English name follow the official list of Brazilian Committee of Ornithological Records (CBRO, version: January 2011).

| ____________________________________________________________________________________________________________________________________________________________ | | | | | | |  |  | | |  |
| --- | --- | --- | --- | --- | --- | --- | --- | --- | --- | --- | --- |
|  |  |  |  |  |  |  | | |  |  | |
| **Family / Scientific name*** | **Location (areas in forest islands and in the continuous forest)** | **D** | **Soc.** | **Habitat** | **Ab** | **English name*** | | |  |  | |
|  |  |  |  |  |  |  | | |  |  | |
| TINAMIDAE |  |  |  |  |  |  | | |  |  | |
| *Tinamus major* | 64, 70 | f | s | F, SF | c | Great Tinamou | | |  |  | |
| *Tinamus* spp. | 64 | o | s | F | r |  | | |  |  | |
| *Crypturellus soui* | 6,10,63,17-1,17-2,18,21,22,23,32,40,41,42,59,64,65,66,69,71 | f | s | F, SF | c | Little Tinamou | | |  |  | |
| *Crypturellus* spp. | 4 | o | s | S, F, SF | r |  | | |  |  | |
|  |  |  |  |  |  |  | | |  |  | |
| CRACIDAE |  |  |  |  |  |  | | |  |  | |
| *Ortalis motmot* | 8, 40, 66, | f | s, C, G | F, SF | r | Variable Chachalaca | | |  |  | |
| *Penelope superciliaris* | 4, 6, 9, 17-1, 17-2, 21, 32, 41, 42, 64, 66 | f | s, C | F, SF | r | Rusty-margined Guan | | |  |  | |
|  |  |  |  |  |  |  | | |  |  | |
| CATHARTIDAE |  |  |  |  |  |  | | |  |  | |
| *Cathartes melambrotus* | 42 | o | s, C | F | c | Greater Yellow-headed Vulture | | |  |  | |
|  |  |  |  |  |  |  | | |  |  | |
| ACCIPITRIDAE |  |  |  |  |  |  | | |  |  | |
| *Accipiter striatus* | 6,65 | o | s | F, SF | r | Sharp-shinned Hawk | | |  |  | |
| *Geranoaetus albicaudatus* | 17-1, 17-2, 41, 65, 70 | o | s | F, SF | c | White-tailed Hawk | | |  |  | |
| *Rupornis magnirostris* | 6, 22, 59, 65 | o | s | S, F, SF | c | Roadside Hawk | | |  |  | |
| *Gampsonyx swansonii* | 17-2 | o | s | F, SF | r | Pearl Kite | | |  |  | |
| *Harpagus bidentatus* | 64 | o | s | F | r | Double-toothed Kite | | |  |  | |
|  |  |  |  |  |  |  | | |  |  | |
| FALCONIDAE |  |  |  |  |  |  | | |  |  | |
| *Falco femoralis* | 22 | o | s, C | F, SF | r | Aplomado Falcon | | |  |  | |
| Herpetotheres cachinnans | 40 | o | s | F, SF | r | Laughing Falcon | | |  |  | |
|  |  |  |  |  |  |  | | |  |  | |
| ODONTOPHORIDAE |  |  |  |  |  |  | | |  |  | |
| *Odontophorus gujanensis* | 71 | o | s, C, G | F, SF | r | Marbled Wood-Quail | | |  |  | |
|  |  |  |  |  |  |  | | |  |  | |
| COLUMBIDAE |  |  |  |  |  |  | | |  |  | |
| *Patagioenas speciosa* | 20, 23, 41, 66 | f | s, C | F, SF | r | Scaled Pigeon | | |  |  | |
| Patagioenas cayennensis | 7, 9, 10, 17-2, 22, 23, 69 | f | s, C | S, F, SF | c | Pale-vented Pigeon | | |  |  | |
| *Claravis pretiosa* | 7, 10, 40, 64, 65 | f | s, C | F, SF | r | Blue Ground-Dove | | |  |  | |
| *Leptotila rufaxilla* | 4, 6, 7, 9, 10, 63,17-2, 21, 22,32, 59, 64, 65, 66, 71 | f | s, C | S, F, SF | c | Gray-fronted Dove | | |  |  | |
| *Zenaida auriculata* | 6 | f | s,C, G | S, F, SF | r | Eared Dove | | |  |  | |
|  |  |  |  |  |  |  | | |  |  | |
| PSITTACIDAE |  |  |  |  |  |  | | |  |  | |
| *Orthopsittaca manilata* | 66, 70 | f | s, C, G | F, SF | c | Red-bellied Macaw | | |  |  | |
| *Aratinga leucophthalma* | 4, 40, 42, 69 | f | C, G | F, SF | c | White-eyed Parakeet | | |  |  | |
| *Aratinga aurea* | 6, 59 | f | C, G | S, F, SF | c | Peach-fronted Parakeet | | |  |  | |
| *Pyrrhura picta* | 65, 70 | f | C, G | F | r | Painted Parakeet | | |  |  | |
| *Brotogeris versicolurus* | 32, 64, 69 | f | G | S, F, SF | c | Canary-winged Parakeet | | |  |  | |
| *Pionites leucogaster* | 64 | f | C, G | F | r | White-bellied Parrot | | |  |  | |
| *Pionus menstruus* | 4, 10, 17-1,18, 40, 41, 64, 65, 66, 69, 71 | f | C, G | F | c | Blue-headed Parrot | | |  |  | |
| *Amazona farinosa* | 65, 66 | f | s, C, G | F, SF | c | Mealy Parrot | | |  |  | |
| *Amazona ochrocephala* | 66, 71 | f | s, C, G | F, SF | c | Yellow-crowned Parrot | | |  |  | |
|  |  |  |  |  |  |  | | |  |  | |
| CUCULIDAE |  |  |  |  |  |  | | |  |  | |
| *Piaya cayana* | 4, 6, 8, 63, 17-2, 18, 23, 32, 41, 66 | o | s | F, SF | c | Squirrel Cuckoo | | |  |  | |
| *Crotophaga major* | 6 | o | C,G | F, SF | c | Greater Ani | | |  |  | |
|  |  |  |  |  |  |  | | |  |  | |
| STRIGIDAE |  |  |  |  |  |  | | |  |  | |
| *Megascops choliba* | 8, 17-2, 41, 59, 65 | o | s, C | F, SF | c | Tropical Screech-Owl | | |  |  | |
| *Asio stygius* | 18, 32, | o | s, C | S, F, SF | c | Stygian Owl | | |  |  | |
| *Strix virgata* | 64 | o | s, C | F | r | Mottled Owl | | |  |  | |
|  |  |  |  |  |  |  | | |  |  | |
| NYCTIBIIDAE |  |  |  |  |  |  | | |  |  | |
| *Nyctibius griseus* | 17-2 | o | s | F, SF | r | Common Potoo | | |  |  | |
|  |  |  |  |  |  |  | | |  |  | |
| CAPRIMULGIDAE |  |  |  |  |  |  | | |  |  | |
| *Hydropsalis albicollis* | 9, 18, 40, 41, 42 | o | s | S, F, SF | c | Pauraque | | |  |  | |
| *Hydropsalis nigrescens* | 4, 40 | o | C, G | F, SF | c | Blackish Nightjar | | |  |  | |
| *Antrostomus rufus* | 6, 17-2, 18, 59 | o | s | F, SF | c | Rufous Nightjar | | |  |  | |
| Hydropsalis torquata | 22 | o | s | S, F, SF | c | Scissor-tailed Nightjar | | |  |  | |
|  |  |  |  |  |  |  | | |  |  | |
| TROCHILIDAE |  |  |  |  |  |  | | |  |  | |
| *Phaethornis superciliosus* | 7, 9, 63, 17-1, 21, 23, 40, 64, 69 | n | s | F, SF | c | Long-tailed Hermit | | |  |  | |
| *Phaethornis bourcieri* | 10, 18, 21, 41, 59, 65, 66, 69 | n | s | F, SF | r | Straight-billed Hermit | | |  |  | |
| *Phaethornis ruber* | 42, 70 | n | s | F | r | Reddish Hermit | | |  |  | |
| *Chlorostilbon notatus* | 69 | n | s | F | r | Blue-chinned Sapphire | | |  |  | |
| Thalurania furcata | 32, 59 | n | s | F, SF | c | Fork-tailed Woodnymph | | |  |  | |
| Hylocharis sapphrina | 18 | n | s | F, SF | r | Rufous-throated Sapphire | | |  |  | |
| Amazilia fimbriata | 63, 17-2, 20, 41, 42, 59, 65, 70 | n | s | F, SF | c | Glittering-throated Emerald * | | |  |  | |
| *Anthracothorax nigricollis* | 21 | n | s | F | r | Black-throated Mango | | |  |  | |
| Eupetomena macroura | 4, 8, 42, 65, 66, 71 | n | s | S, F, SF | c | Swallow-tailed Hummingbird | | |  |  | |
| Glaucis hirsutus | 6, 17-1 | n | s | F, SF | r | Rufous-breasted Hermit | | |  |  | |
| *Polytmus theresiae* | 20, 32, 66 | n | s | F, SF | r | Green-tailed Goldenthroat | | |  |  | |
|  |  |  |  |  |  |  | | |  |  | |
| TROGONIDAE |  |  |  |  |  |  | | |  |  | |
| *Trogon viridis* | 4, 6, 7, 9, 10, 17-1, 17-2, 32, 40, 41, 63, 64, 65, 66, 69, 70, 71 | f | s, C | F, SF | c | White-tailed Trogon | | |  |  | |
|  |  |  |  |  |  |  | | |  |  | |
| MOMOTIDAE |  |  |  |  |  |  | | |  |  | |
| *Momotus momota* | 4, 9, 10, 17-1,23, 32, 41, 42, 63, 64, 66, 69, 70, 71 | o | s | F | r | Amazonian Motmot | | |  |  | |
|  |  |  |  |  |  |  | | |  |  | |
| BUCCONIDAE |  |  |  |  |  |  | | |  |  | |
| *Notharchus tectus* | 6, 41, 65, 71 | o | s, C | F | r | Pied Puffbird | | |  |  | |
| *Nystalus maculatus* | 59 | o | s, C, G | S, F | c | Spot-backed Puffbird | | |  |  | |
| *Monasa nigrifrons* | 64 | o | s, C, G | F, SF, A | c | White-fronted Nunbird | | |  |  | |
| *Chelidoptera tenebrosa* | 41 | o | s, C | F, SF | c | Swallow-winged Puffbird | | |  |  | |
|  |  |  |  |  |  |  | | |  |  | |
| RAMPHASTIDAE |  |  |  |  |  |  | | |  |  | |
| *Pteroglossus aracari* | 4, 32 | f | G | F, SF | r | Black-necked Aracari | | |  |  | |
| *Pteroglossus inscriptus* | 17-2, 42, 69 | f | G | F, SF | r | Lettered Aracari | | |  |  | |
| *Ramphastos vitellinus* | 4, 6, 17-2, 18, 20, 23, 32, 40, 41, 42, 64, 65, 66, 69, 70, 71 | f | s, C, G | F, SF | r | Channel-billed Toucan | | |  |  | |
|  |  |  |  |  |  |  | | |  |  | |
| PICIDAE |  |  |  |  |  |  | | |  |  | |
| *Veliniornis affinis* | 6, 7, 9, 20, 32, 41, 65, 69 | o | s | F | r | Red-stained Woodpecker | | |  |  | |
| *Piculus flavigula* | 7, 32, 66 | o | s | F | r | Yellow-throated Woodpecker | | |  |  | |
| *Picumnus aurifrons* | 64 | o | s | F | r | Bar-breasted Piculet | | |  |  | |
| *Dryocopus lineatus* | 10, 63, 17-1, 18, 21, 32, 40, 41, 64, 65, 66, 69, 70 | o | s | F | r | Lineated Woodpecker | | |  |  | |
| *Campephilus rubricollis* | 4 | o | s | F | r | Red-necked Woodpecker | | |  |  | |
|  |  |  |  |  |  |  | | |  |  | |
| THAMNOPHILIDAE |  |  |  |  |  |  | | |  |  | |
| *Thamnophilus stictocephalus* | 4,5,9,10,17-1,17-2,18,20,21,22,23,32,40,41,42,59,63,64,65,66,69,71 | o | s, C | F, SF | c | atterer's Slaty-Antshrike | | |  |  | |
| *Thamnomanes caesius* | 65 | o | s, C | F | c | Cinereous Antshrike | | |  |  | |
| *Myrmotherula axillaris* | 4, 17-2, 21, 40, 42, 64, 65, 66, 69, 71 | o | s, C | F | r | White-flanked Antwren | | |  |  | |
| *Cercomacra cinerascens* | 6, 7, 10, 17-1, 17-2, 18, 20, 22, 41, 42, 64, 66, 71 | o | s, C | F, SF | r | Gray Antbird | | |  |  | |
| *Hypocnemis cantator* | 64, 65, 66, 69, 70 | o | s, C | F | c | Guianan Warbling-Antbird | | |  |  | |
| *Myrmeciza hemimelaena* | 4, 18, 32, 65, 66 | o | s, C | F | c | Chestnut-tailed Antbird | | |  |  | |
| *Phlegopsis nigromaculata* | 66, 71 | o | s, C | F | r | Black-spotted Bare-eye | | |  |  | |
| *Formicivora grisea* | 4, 6, 7, 8, 9, 10, 17-2, 18, 20, 21, 22, 23, 32, 40, 41, 42, 59, 63, 64, 65, 65, 69, 70, 71 | o | s, C | F | c | White-fringed Antwren | | |  |  | |
| *Formicivora rufa* | 20 | o | s, C | S, F, SF | c | Rusty-backed Antwren | | |  |  | |
|  |  |  |  |  |  |  | | |  |  | |
| FORMICARIIDAE |  |  |  |  |  |  | | |  |  | |
| *Formicarius colma* | 64, 66, 70, | o | s, C | F | r | Rufous-capped Antthrush | | |  |  | |
|  |  |  |  |  |  |  | | |  |  | |
| DENDROCOLAPTIDAE |  |  |  |  |  |  | | |  |  | |
| *Dendrocincla fuliginosa* | 9, 17-1, 21, 40, 42, 63, 64, 65, 66, 70, 71 | o | s | F, | c | Plain-brown Woodcreeper | | |  |  | |
| *Sittasomus griseicapillus* | 4, 17-1, 23, 64, 65, 66, 69 | o | s | F, SF | c | Olivaceous Woodcreeper | | |  |  | |
| *Glyphorynchus spirurus* | 4, 65 | o | s | F | c | Wedge-billed Woodcreeper | | |  |  | |
| *Lepidocolaptes angustirostris* | 7,8, 40, 41, 59, 69 | o | s | S, F, SF | c | Narrow-billed Woodcreeper | | |  |  | |
| *Dendroplex picus* | 6, 7, 9, 10, 17-1, 17-2, 18, 20, 21, 23,32, 63, 64, 65, 66, 69, 70, 71 | o | s | F, SF | c | Straight-billed Woodcreeper | | |  |  | |
| *Xiphorhynchus guttatus* | 4, 65, 66 | o | s | F, SF | c | Buff-throated Woodcreeper | | |  |  | |
| *Dendrocolaptes certhia* | 6, 42, 64, 66 | o | s | F | r | Amazonian Barred-Woodcreeper | | |  |  | |
| *Campyloramphus procurvoides* | 64 | o | s | F | c | Curve-billed Scythebill | | |  |  | |
|  |  |  |  |  |  |  | | |  |  | |
| FURNARIIDAE |  |  |  |  |  |  | | |  |  | |
| *Synallaxis rutilans* | 9, 10, 17-1, 17-2, 18, 21, 22, 23, 32, 41, 42, 63, 64, 65, 69, 70, 71 | o | s, C | F, SF | c | Ruddy Spinetail | | |  |  | |
| *Xenops minutus* | 6, 10, 23, 42, 66, 69 | o | s | F | r | Plain Xenops | | |  |  | |
|  |  |  |  |  |  |  | | |  |  | |
| PIPRIDAE |  |  |  |  |  |  | | |  |  | |
| *Dixiphia pipra* | 4, 6, 32 | f | s | F | c | White-crowned Manakin | | |  |  | |
| *Chiroxiphia pareola* | 4,6,8,9,10,17-1, 17-2, 18,20, 21, 22, 23, 32, 40, 41, 42,63, 64, 65, 66, 69, 70, 71 | f | s, C, G | F | c | Blue-backed Manakin | | |  |  | |
| *Manacus manacus* | 4, 6, 10, 17-1,18, 21, 22, 41, 63, 64, 70, 71 | f | s, C | F | c | White-bearded Manakin | | |  |  | |
| *Neopelma palescens* | 7, 23, 59, 69 | o | s | F | r | Pale-bellied Tyrant-Manakin | | |  |  | |
| *Tyranneutes stolzmanni* | 4, 17-1, 18, 32, 40, 65, 69, 70, 71 | o | s | F | r | Dwarf Tyrant-Manakin | | |  |  | |
|  |  |  |  |  |  |  | | |  |  | |
| TYTIRIDAE |  |  |  |  |  |  | | |  |  | |
| *Pachyramphus rufus* | 17-2, 32, 40, 42, 64, 70 | o | s, C | F, SF | r | Cinereous Becard | | |  |  | |
| *Tityra inquisitor* | 17-1, 18, 65, 70, 71 | o | s, C | F, SF | r | Black-crowned Tityra | | |  |  | |
| *Terenotriccus erythrurus* | 69 | o | s | F | r | Ruddy-tailed Flycatcher | | |  |  | |
|  |  |  |  |  |  |  | | |  |  | |
| COTINGIDAE |  |  |  |  |  |  | | |  |  | |
| *Lipaugus vociferans* | 40, 64, 65, 66, 70 | o | s | F | c | Screaming Piha | | |  |  | |
|  |  |  |  |  |  |  | | |  |  | |
| TYRANNIDAE |  |  |  |  |  |  | | |  |  | |
| *Camptostoma obsoletum* | 6, 8, 10, 17-1, 17-2, 20, 21, 23, 40, 42, 63, 69 | o | s | F, SF | c | Southern Beardless-Tyrannulet | | |  |  | |
| *Tyrannulus elatus* | 10, 17-1, 17-2, 18, 20, 40, 41, 42, 59, 63, 69 | o | s, C | F, SF | c | Yellow-crowned Tyrannulet | | |  |  | |
| *Myiopagis gaimardii* | 4, 9, 10, 17-1, 17-2, 18, 21, 23, 32, 41, 42, 63, 64, 65, 66, 69, 70 | o | s, C | F | r | Forest Elaenia | | |  |  | |
| *Suiriri suiriri* | 7, 66, 69 | o | s, C, G | S, F, SF | c | Suiriri Flycatcher | | |  |  | |
| *Elaenia chiriquensis* | 6, 20, 21, 40, 41, 59, 63, 64 | o | s, C | S,F,SF,A | c | Lesser Elaenia | | |  |  | |
| *Elaenia cristata* | 6, 7, 8, 9, 20, 21, 22, 23, 40, 42, 59, 63, 64, 69 | o | s, C | S, F, SF | c | Plain-crested Elaenia | | |  |  | |
| *Elaenia flavogaster* | 22, 32, 41, 63, 66, 69 | o | s, C | S, F, SF | r | Yellow-bellied Elaenia | | |  |  | |
| *Mionectes macconnelli* | 40, 66, 70 | o | s, C | F | c | McConnell's Flycatcher | | |  |  | |
| *Hemitriccus striaticollis* | 4, 6, 7, 8, 9, 10, 17-1, 17-2, 18, 20, 23, 32, 41, 42, 64, 66, 70, 71 | o | s | F | c | sebinho-rajado-amarelo | | |  |  | |
| *Todirostrum cinereum* | 17-1, 17-2, 18, 21, 22, 23, 32, 40, 41, 42, 59, 64, 65, 66, 70 | o | s | F, SF | c | Common Tody-Flycatcher | | |  |  | |
| *Tolmomyias flaviventris* | 4, 6, 7, 8, 9, 10, 1, 17-1, 17-2, 18, 20, 21, 22, 23, 32, 40, 41, 42, 59, 64, 65, 66, 69, 71 | o | s | F, SF | c | Yellow-breasted Flycatcher | | |  |  | |
| *Phaeomyias murina* | 17-2, 63, 66, 71 | o | s | F, SF | r | Mouse-colored Tyrannulet | | |  |  | |
| *Attila spadiceus* | 17-1, 32, 64, 66, 69, 70, 71 | o | s | F | r | Bright-rumped Attila | | |  |  | |
| *Myiarchus ferox* | 6, 17-1, 17-2, 23, 40, 42 | o | s, C | S,F,SF,A | c | Short-crested Flycatcher | | |  |  | |
| *Myiarchus* spp. | 8, 21, 22 | o | s | F, SF | r |  | | |  |  | |
| *Myiarchus tyrannulus* | 7, 8, 17-2, 21, 22, 23, 40, 42, 59, 66, 71 | o | s, C | S,F,SF,A | r | Brown-crested Flycatcher | | |  |  | |
| *Pitangus sulphuratus* | 7, 59, 69 | o | s, C | F, SF, A | c | Great Kiskadee | | |  |  | |
| *Megarynchus pitangua* | 7, 17-2, 18, 21, 40, 42, 64 | o | s | F, SF | r | Boat-billed Flycatcher | | |  |  | |
| *Myiozetetes cayenensis* | 6, 22, 23, 59 | o | s, C, G | F, SF | c | Rusty-margined Flycatcher | | |  |  | |
| *Myiodynastes maculatus* | 17-1, 17-2, 20, 66, 69 | o | s | F, SF | r | Streaked Flycatcher | | |  |  | |
| *Legatus leucophaius* | 17-1, 17-2, 18, 20, 32, 40, 42, 64, 65, 69 | o | s | F, SF | c | Piratic Flycatcher | | |  |  | |
| *Empidonomus varius* | 8, 59, 66 | o | s | F, SF | c | Variegated Flycatcher | | |  |  | |
| *Tyrannus melancholicus* | 6, 63, 17-1, 18, 40, 59, 69 | o | s, C | S, F, SF, A | c | Tropical Kingbird | | |  |  | |
| *Tyrannus albogularis* | 4, 7, 22, 59, 66 | o | s, C | S,F,SF,A | c | White-throated Kingbird | | |  |  | |
| *Sirystes sibilator* | 17-2, 64 | o | s | S, F, SF | r | Sirystes | | |  |  | |
|  |  |  |  |  |  |  | | |  |  | |
| VIREONIDAE |  |  |  |  |  |  | | |  |  | |
| *Vireo olivaceous* | 4,6, 7, 8, 9, 10,17-1,17-2,20,21, 23, 32, 41, 42, 59, 63, 64, 65, 66, 69, 70 | o | s | S, F, SF | c | Red-eyed Vireo | | |  |  | |
| *Cyclarhis gujanensis* | 6, 7, 8, 20, 21, 23, 40, 63, 70, 71 | o | s | S,F,SF,A | c | Rufous-browed Peppershrike | | |  |  | |
| Hylophilus pectoralis | 4, 7, 8, 9, 17-2, 18, 20, 21, 23 32, 40, 42, 59, 63, 64, 69 | o | s | F, SF | r | Ashy-headed Greenlet | | |  |  | |
|  |  |  |  |  |  |  | | |  |  | |
| HIRUNDINIDAE |  |  |  |  |  |  | | |  |  | |
| *Progne chalybea* | 6 |  | C, G | F | c | Gray-breasted Martin | | |  |  | |
|  |  | o |  |  |  |  | | |  |  | |
| TROGLODYTIDAE |  |  |  |  |  |  | | |  |  | |
| *Cantorchilus leucotis* | 4,6,7,8,9,10, 17-1, 17-2, 18, 20, 21, 22, 23, 32, 40, 41, 42, 59, 64, 66, 69, 70, 71 | o | C | F, SF, A | c | Buff-breasted Wren | | |  |  | |
| *Troglodytes musculus* | 8 | o | s, C | S,F,SF,A | c | Southern House Wren | | |  |  | |
|  |  |  |  |  |  |  | | |  |  | |
| TURDIDAE |  |  |  |  |  |  | | |  |  | |
| *Turdus leucomelas* | 4, 6,7, 8, 9, 17-1,17-2,20, 22, 23, 32, 40, 41, 42,59, 63, 64, 69, 70 | f | s | F, SF | c | Pale-breasted Thrush | | |  |  | |
|  |  |  |  |  |  |  | | |  |  | |
| THRAUPIDAE |  |  |  |  |  |  | | |  |  | |
| *Saltator coerulescens* | 41 | f | s, C | S, F, SF | r | Grayish Saltator | | |  |  | |
| *Tachyphonus rufus* | 4,18,40, 69, 71 | o | s, C | F, SF | r | White-lined Tanager | | |  |  | |
| *Ramphocelus carbo* | 4, 6, 8, 9, 17-1, 17-2, 18, 66, 69 | o | s, C, G | F, SF, A | c | Silver-beaked Tanager | | |  |  | |
| *Thraupis episcopus* | 6, 7, 8, 9, 10, 17-1, 18, 20, 21, 22, 32, 40, 41, 63, 69 | f | s, C | S,F,SF,A | c | Blue-gray Tanager | | |  |  | |
| *Thraupis palmarum* | 17-2, 21, 32, 41, 64, 65, 66, 71 | f | s, C | F, SF, A | c | Palm Tanager | | |  |  | |
| *Tangara cayana* | 7, 17-2, 22, 23, 42, 59, 63, 64, 71 | f | s, C, G | S, F, SF | c | Burnished-buff Tanager | | |  |  | |
| *Tangara mexicana* | 64 | f | s, C | F | r | Turquoise Tanager | | |  |  | |
| *Dacnis cayana* | 4, 8, 17-1, 17-2, 18, 41, 66, 69, 71 | f | s, C | S, F, SF | r | Blue Dacnis | | |  |  | |
| *Dacnis flaviventer* | 69 | f | s, C | F | r | Yellow-bellied Dacnis | | |  |  | |
| *Cyanerpes cyaneus* | 7, 9, 18, 65, 69 | f | s, C | F | r | Red-legged Honeycreepe | | |  |  | |
| *Nemosia pileata* | 17-2, 18, 21, 41, 69 | o | s, C, G | F, SF | r | Hooded Tanager | | |  |  | |
| *Schistochlamys melanopis* | 21 | f | s | S, F, SF | r | Black-faced Tanager | | |  |  | |
|  |  |  |  |  |  |  | | |  |  | |
| EMBERIZIDAE |  |  |  |  |  |  | | |  |  | |
| *Sporophila angolensis* | 6 | f | s, C | F, SF | r | Chestnut-bellied Seed-Finch | | |  |  | |
|  |  |  |  |  |  |  | | |  |  | |
| CARDINALIDAE |  |  |  |  |  |  | | |  |  | |
| *Cyanoloxia cyanoides* | 42, 66, 71 | f | s, C | F, SF | r | Blue-black Grosbeak | | |  |  | |
|  |  |  |  |  |  |  | | |  |  | |
| PARULIDAE |  |  |  |  |  |  | | |  |  | |
| *Phaeothlypis rivularis* | 70 | o | s | F | r | Neotropical River Warbler | | |  |  | |
|  |  |  |  |  |  |  | | |  |  | |
| ICTERIDAE |  |  |  |  |  |  | | |  |  | |
| *Psarocolius decumanus* | 17-2, 18, 42, 66, 69, 70, 71 | o | s, C, G | F, SF | c | Crested Oropendola | | |  |  | |
| *Cacicus cela* | 32, 66, 69, 70, 71 | o | s, C, G | F, SF, A | c | Yellow-rumped Cacique | | |  |  | |
| Icterus jamacaii | 3, 71 | o | s | F | r | Campo Troupial | | |  |  | |
|  |  |  |  |  |  |  | | |  |  | |
| FRINGILLIDAE |  |  |  |  |  |  | | |  |  | |
| *Euphonia chlorotica* | 17-1, 21, 23, 32, 40, 41, 59, 64, 69, 71 | f | s, C | S, F, SF | r | Purple-throated Euphonia | | |  |  | |
|  |  |  |  |  |  |  | | |  |  | |

__________________________________________________________________________________________________________________ **Location:** 4 – 59 are forest island sites, and the remaining areas 64 to 71 are in the continuous forest; Codes: **Soc.= Sociality:** **s** = solitary; **C**= couple; **G** = in groups or small flocks; **Habitat:** **F** = Terra firme forest; **SF**= Secondary Forest; **A** = deforested areas. **Ab=Abundance in Alter-do-Chão: c** = common; **r** = rare). **D** = Diet,  **f =** frugivore; **o=** omnivore- insectivore; Diet type were based on informations available in Schubart *et al.* (1965) and complemented with direct field observations by the first author of this study.
