# Supplementary material for: Spatial and temporal changes in bird assemblages in forest fragments in an eastern Amazonian savannah
Source: Ecol Evol. 2013 Aug 6;3(10):3249–62. doi: 10.1002/ece3.700 (PMC3797474; doi:10.1002/ece3.700)
Supplement: Supplementary file 2 [file ece30003-3249-SD2.doc]

**Appendix S2. Analysis on bird guilds**

Depending on the amount of variance explained by the MDS-axis we used two dimensional solutions (two MDS-axes together, because the amount of variance they explained was at least near to 70 %, i.e for omnivores and frugivores) as a dependent variable in some models of a multivariate analysis of variance (MANOVA) to investigate whether the bird community composition differed among the following forest fragments categories: s = small i.e. 1 to 15 ha; m = medium or 20 to 49 ha; L = large greater than 50 ha; and between these fragment categories and the areas in the continuous forest.

# Differences in bird community (guilds) composition among forest fragments of difference sizes, and between them and the continuous forest

The MDS ordination results indicated that, for the whole community, omnivores and frugivores (considering both, the quantitative or abundance, and the qualitative or presence/absence data of species by site), the differences in bird species composition was significant among forest fragment sizes and between them and the areas of continuous forest (Fig. 1, Table 1). The small forest fragment sites (“S” symbols) were always clustered apart from the other fragment sizes (and mostly in the first dimension or MDS1) in the diagrams, and this was also true for the areas of continuous forest (symbol “C” in Fig. 1). Because only three areas of continuous forest were sampled in 1999, we did not run much analysis for that year. However, even with the small sample size in 1999, the bird species composition (using qualitative data) also differed significantly in forest fragment sites when compared to continuous forest (Manova, Pillai’s Trace = 0.285, F_2,19_ = 3.788, p = 0.041).

Although the pattern of clustering of the areas was not similar in each graph (sometimes separating groups of the same letters in the first MDS dimension and sometimes in the second), the overall pattern was similar, independently of the bird group considered. The bird communities of medium forest fragments were intermediate between large and small forest fragments. The avifauna of medium size forest fragments was also more similar among them than was found when comparing them with small and large fragments. One of the reasons for this could be that medium size fragments may have similar densities of very territorial species, such as *Formicivira grisea*, *Thamnophilus stictocephalus* and *Cantorchilus leucotis*, and these may therefore limit the number of other species that can colonize the areas (see below). These birds are mainly insectivores and seem to prefer areas bearing a relatively complex understorey forest structure; their density in the large fragments was particularly high, perhaps because large fragments tend to have a more open understorey due to a lower proportion between fragment size and the forest edge perimeter.

Table 1. Results of multivariate analysis of variance (MANOVA) comparing the similarities of bird community composition among forest fragment size and between the fragments and the areas of continuous forest (see also Fig. 1), on the savannahs of Alter-do-Chão, Pará, eastern Brazilian Amazon.

____________________________________________________________________________________________________ Quantitative community composition Qualitative community composition

Pillai’s Trace df F p Pillai’s Trace df F p

Entire bird community 1.011 6, 42 7.162 0.0001 0.757 6, 47 4.267 0.002

Omnivores–insectivores 0.845 6, 42 5.119 0.001 0.748 6, 42 4.187 0.002

Frugivores 0.515 6, 42 2.429 0.042 0.508 6, 42 2.386  0.045

Figure 1. Results from the Multidimensional Scaling ordination analysis (MDS) used to generate a simple ordination of areas (forest fragments and areas in the continuous forest) in the savannahs of Alter-do-Chão, based on the similarity matrix of distances generated by the Bray–Curtis quantitative index (graphics on the left) and Canberra qualitative (presence/absence) index (graphics on right). Results of MDS include: 1) the whole quantitative community, in which the abundance of each species was included in the analysis (upper graphic on left); 2) the omnivore/insectivore guild (middle graphic on left); 3) the frugivore guild (lower graphic on left). Results of MDS include: 1) the whole qualitative community, in which only the presence or absence of species was included in the analysis (upper graphic on right); 2) the omnivore/insectivore guild (middle graphic on right); 3) the frugivore guild (lower graphic on right). In the figures the letters represent: S = small forest fragments (1 to 9 ha); M = medium size forest fragments (10 to 49 ha); L = large forest fragments (50 ha and 340 ha); C = continuous forest. Each letter corresponds to a sampled area (see Appendix 1 for characteristics of the areas). The closer the letters (areas) on the figure, the more similar are the bird communities in the species composition of the areas. Bird community composition was more similar among forest fragments than between the fragments and the areas of continuous forest. The sample unit in each area was an area of 3.7 hectares (see Methods). See also Table 1 for MANOVA results comparing MDS among the areas.

Fig.1
